# How Personality Affects Vulnerability among Israelis and Palestinians following the 2009 Gaza Conflict

**DOI:** 10.1371/journal.pone.0156278

**Published:** 2016-07-08

**Authors:** Daphna Canetti, Shaul Kimhi, Rasmiyah Hanoun, Gabriel A. Rocha, Sandro Galea, Charles A. Morgan

**Affiliations:** 1 School of Political Science, University of Haifa, Haifa, Israel; 2 Department of Psychology, Tel-Hai College, Tel-Hai, Israel; 3 Faculty of Educational Science, An-Najah National University, Nablus, Palestine; 4 Carolinas Biofeedback Clinic, Charlotte, North Carolina, United States of America, and Doctors Making Housecalls, Durham, North Carolina, United States of America; 5 School of Public Health, Boston University, Boston, Massachusetts, United States of America; 6 National Security Program, University of New Haven, New Haven, Connecticut, United States of America, and School of Medicine, Yale University, New Haven, Connecticut, United States of America; University of Stellenbosch, SOUTH AFRICA

## Abstract

Can the onset of PTSD symptoms and depression be predicted by personality factors and thought control strategies? A logical explanation for the different mental health outcomes of individuals exposed to trauma would seem to be personality factors and thought control strategies. Trauma exposure is necessary but not sufficient for the development of PTSD. To this end, we assess the role of personality traits and coping styles in PTSD vulnerability among Israeli and Palestinian students amid conflict. We also determine whether gender and exposure level to trauma impact the likelihood of the onset of PTSD symptoms. Five questionnaires assess previous trauma, PTSD symptoms, demographics, personality factors and thought control strategies, which are analyzed using path analysis. Findings show that the importance of personality factors and thought control strategies in predicting vulnerability increases in the face of political violence: the higher stress, the more important the roles of personality and thought control strategies. Thought control strategies associated with introverted and less emotionally stable personality-types correlate positively with higher levels of PTSD symptoms and depression, particularly among Palestinians. By extension, because mental health is key to reducing violence in the region, PTSD reduction in conflict zones warrants rethinking.

## Introduction

Posttraumatic stress disorder (PTSD) resulting from exposure to conflict increases threat perceptions, contributing to support for militant policies [1]. While it is widely accepted and known that exposure to traumatic events leads to PTSD, there are several theories that suggest different mechanisms for this relationship. One mechanism is resources, according to the Conservation of Resources Theory (COR) the loss or gain of both personal and material resources may affect an individuals’ likelihood to experience PTSD [2]. COR theory suggests that the influence of any stress on an individual depends primarily on the threat of loss and / or real loss of resources. The theory talks about four categories of resources: material, personal, resources which are connected to living conditions, and “energy” resources such as money and knowledge. Other studies have also shown that cognitive function, appraisal [3] and threat perceptions [4] are additional mechanisms that have been used to explore the relationship between exposure to traumatic events and PTSD. Besser et al. [5] found personality to help explain the association between an individual’s PTSD and the severity of their trauma exposure.

The current study expands on these theories to examine how various mechanisms such as to what degree personality factors and thought control strategies play a role in determining whether an individual exposed to traumatic events will experience PTSD and/or depression symptoms. Furthermore, it is understood that individual circumstances may alter the extent to which these mechanisms apply and the extent to which individuals may experience PTSD and/or depression symptoms. This study therefore uses exposure to traumatic events from Gaza 2008–2009 to compare how Palestinians and Israelis—exposed to the same conflict but having different socio-economic status—differ from one another in each aspect of our theory.

Traumatic events’ negative psychological effects have been discussed in several studies [6–8]. Reliving, or being unable to reconcile these events, over a long period of time may cause PTSD [9–11], as well as emotional and behavioral problems. We examine PTSD symptoms and depression among Palestinian and Israeli students who live under stress from exposure to traumatic events from the ongoing conflict between these two nations.

While exposure to trauma may lead to psychopathology and ill-health for some, others show resilience [12–14]. The variability among peoples' ability to withstand potential traumatic events, PTE [15], may be due to the interplay between genetic, biological processes and different environments [16–18]. In order to examine differences and similarities between Palestinian and Israeli students we focus on one situational characteristic (level of exposure) and two individual characteristics (two of the Big Five personality factors and five thought control strategies) as predictors of PTSD symptoms and depression which share comorbidity.

### Exposure to traumatic experience

Several studies have shown that war and ongoing political violence are associated with higher levels of PTSD symptoms and depression [19–26]. In low-income countries, such as in the West Bank, the impact of political violence on mental health may be exacerbated by deteriorating economic conditions, education, social services, routine functioning of government, availability of food, services, and industry [22, 23]. This echoes Conservation of Resources theory which argues that the internal (i.e. self-esteem) and external resources of an individual may mitigate levels of PTSD [2]. The Palestinian students represent such a population.

### Israelis and Palestinians in the range of fire

The Israeli-Palestinian conflict imposes high material and psychological demands [27, 28]. It is asymmetrical [29]; characterized by a large power imbalance in favor of the Israeli state [30], and a reality of structured inequalities [31]. Israelis suffer solely from Palestinian violence; Palestinians are also victims of violence from inter-factional clashes [32–34].

The daily stresses Palestinians face [35], just as the need for Israelis to be ever-vigilant against rocket attacks, takes its toll on cognitive and physical abilities [36]. Civilians exposed to the effects of political violence, or living in constant threat, collectively suffer from stress symptoms, depression, anxiety, and reduced cognitive function.

The indications that continuous exposure to violence has led to heightened levels of distress and threat perception in both groups are of relevance to the underlying mechanisms in our study [4, 36–38]. A large survey study set in Israel [39] found almost one third of the sample reporting some form of impairment caused by post-traumatic stress, a fifth meeting the full criteria for PTSD. For example, a study in Sderot, an area hard-hit by Palestinian rocket attacks, found 43.5 percent of its seventh and eighth graders displaying clinical signs of PTSD [40]. A study conducted in the Palestinian territories [32] concluded that PTSD and depression in the West Bank, Gaza, and East Jerusalem is extremely high, ranging from 16.1–29.9% of the population. Other studies which examined the level of PTSD among Palestinians [22, 25] indicate a high level of PTSD ranging from 18% to 50% within Palestinian population. These statistics underscore the level of stress and threat engendered by the high levels of violence endemic to the conflict.

### The big five factors, PTSD and depression symptoms

The big five factor model (FFM) of personality [41] states that normal personality traits fall along five dimensions: Openness to experience, conscientiousness, extraversion, agreeableness, and neuroticism—emotional stability vs. instability [41–43]. Most studies which examine negative events and PTSD, focus on the Big Five as predicting PTSD [44], and suggest that the Big Five have a physiological and genetic basis [45].

We used two personality factors: extraversion and emotional stability. Studies which examined the other three personality factors as predicting PTSD and depression have been inconclusive; therefore, they were omitted from this study. Research has indicated that these two personality factors significantly predict PTSD and depression: (a) **Extraversion–**studies show a link between low extraversion and PTSD symptoms [46, 43, 47, 48]. (b) **Neuroticism**—Higher levels of neuroticism are often associated with higher levels of PTSD and depression [49–57].

### Thought control strategies and PTSD symptoms

Cognitive models of PTSD have implicated thought control in the development of, maintenance of, and recovery from, PTSD [58–61]. The Thought Control Questionnaire (TCQ) assesses five types of strategies to cope with negative thoughts: distraction, reappraisal, social control, worry, and punishment. Studies indicate a positive association with PTSD in thought control strategies like worry and self-punishment; distraction, social control, and reappraisal are negatively associated [58, 62–64].

Overall, we assumed that the associations between the two personality factors and the five thought control strategies and PTSD as well as depression will be stronger among Palestinians compared with Israeli students. To the best of our knowledge these assumption have not been researched before. However, based on COR theory we may claim that the Palestinians have lost more external resources (e.g. higher level of exposure [37]) and internal resources (e.g. feelings of humiliations [65]) compared with the Israelis.

### Hypotheses

In accordance with the theory previously presented and as stated above we hypothesize:

H1: Palestinian students will report higher levels of trauma experience, PTSD and depression than Israeli students.
H2: Higher levels of extraversion and emotional stability are hypothesized to predict higher levels of PTSD and depression. These predictions will be stronger among Palestinians than Israelis.
H3: The five thought control strategies will significantly predict levels of PTSD and depression: higher use of worry and punishment will predict higher levels of PTSD and depression. These predictions will be stronger among Palestinians than Israelis.

## Method

### Sampling and data collection

Participants were recruited from universities and responded to questionnaires on previous trauma experiences; PTSD, depression, demographics, personality factors and thought control mechanisms. Universities provide a representative population cross-section. Students in Israel tend to be older than the international norm due to army and national service, providing a representative cross-section of ages; the universities selected were in geographically diverse locations. Data was collected face-to-face following the 2008–9 Gaza conflict. Students were from varying departments (including Humanities, Fine Arts, Political Science and Medicine). *Palestinian* participants (n = 514) were from Al Quds Open University and An-Najah National University (94%). Men comprised 40% of respondents; the mean age was 22.54 (SD = 3.58), family income (compare to average family income in your country) 2.95 (scale 1–5), 17% were married and only 8% reported that they are secular and 25% reported that they are traditional. *Israeli* participants (n = 256) were from Tel Hai College (21%), University of Haifa (40%), Tel Aviv University, and Ashkelon College. Women comprised 32% of the respondents; the mean age was 26 (SD = 4.67). Family income was 3.08, 17% were married and only 68% reported that they are secular and 14% reported that they are traditional. The Yale University Institutional Review Board and the Haifa University Institutional Review Board approved this study.

### Measures

#### Independent variables

Trauma Inventory (Brief Trauma Questionnaire). The Brief Trauma Questionnaire (BTQ), developed by Schnurr and colleagues [66], assesses 10 traumatic events. For all endorsed traumatic events, respondents were asked if they thought their lives were in danger, if they thought they might be seriously injured, or if they were injured. The BTQ contains 10 items and on each of the items the person responds three times (1 = yes or 2 = nor): did it occur? Is your life was in danger? Where you injured? The sum of the items reflects BTQ score (range 0–114). The BTQ was originally designed to assess traumatic exposure according to DSM-IV but in this study respondents were specifically asked only about Criterion A.1 (life threat/serious injury). The BTQ has been shown to have good reliability [66, 67].

*Trauma questionnaire*: The trauma questionnaire is a brief measure of trauma that assesses criterion A1 (DSM IV). The Trauma Questionnaire scale includes the assessment of a wide range of traumatic experiences that are not asked in the BTQ, including age at the time, frequency of occurrence, length of trauma, if more than one trauma was experienced, and the respondent’s worst trauma [67]. Questions 1–3 were taken from the Peritraumatic Distress Inventory (PDI) and questions 4–5 from the Peritraumatic Dissociative Experiences Questionnaire (PDEQ) [68].

This phase concludes with a summary of the coded answers. For each respondent we sum the answers. We add the sum of both scales (BTQ and Trauma Questionnaire) into one score representing traumatic experience.

*Ten Item Personality Inventory (TIPI)*: Gosling’s [69] TIPI is an abbreviated measurement of the Big Five Dimensions of Personality. It begins with the stem “I see myself as: “followed by ten pairs of two trait descriptors, which participants rate on a 7-point Likert-type scale. The results are broken up into five scores; one for each personality dimension. We use only two factors: extraversion and emotional stability. After changing the direction of the opposite items to coincide with each other and adding the two items representing each factor, each student has two scores: higher scores represent greater extraversion and emotional stability, respectively. Overall, results indicate that the BFI-10 scales retain significant levels of reliability and validity [70].

*Thought Control Questionnaire*: A 30-item questionnaire [71] consisting of five subscales and assesses individual differences in the control of intrusive thoughts by strategies of distraction, worry, social control, punishment, and reappraisal, is used, which participants rate on a 4- point scale. The total TCQ score is obtained by summing the individual subscales.

### Dependent variables

#### PTSD

The Posttraumatic stress disorder symptom scale (PSS Total), [72], is a seventeen-item scale designed to assess the 17 PTSD symptoms within the 3 major symptom clusters: re-experiencing, avoidance/numbing, and arousal categories as identified in DSM-IV. The PSS has been found to hold reliable psychometric properties which give it predictive power among Israelis and Palestinians [73]. The PSS provides one score per symptom cluster, and a total symptom count. One question has been added to assess functional impairment. The instrument asks individuals “the extent to which you have experienced each of these feelings in the past month?” Phrases describing the 17 symptoms are given. Individuals are asked to rate their responses using a 4- point scale (1 = not at all, 4 = very much). Responses are summed for each respondent. Cronbach’s Alpha in our study is .92 [72].

#### Depression symptoms

The PHQ-9 is a self-administered version of the PRIME-MD diagnostic instrument for common mental disorders. This instrument scores each of the 9 DSM-IV criteria for depression as: 1-not at all, 2-several days, 3- more than half the days, and 4-nearly every day. We tally each response by the number value under the answer heading, adding the numbers together to get the total score. Total scores are broken up into 3 categories (4 or less, 5–14, and 15 or more). Cronbach’s Alpha in our study is .88.

#### Demographic questionnaire

It assesses gender, age, education level, household income, marital status, religious affiliation, religiosity, place of birth, year of immigration if applicable, place of residence.

## Results

We analyzed our data according to: (a) descriptive statistics, (b) correlations among research variables, (c) MANOVA analysis to examine differences between the groups, (d) path analysis to examine the unique prediction of PTSD and depression by the independent variables.

**Fig 1:**
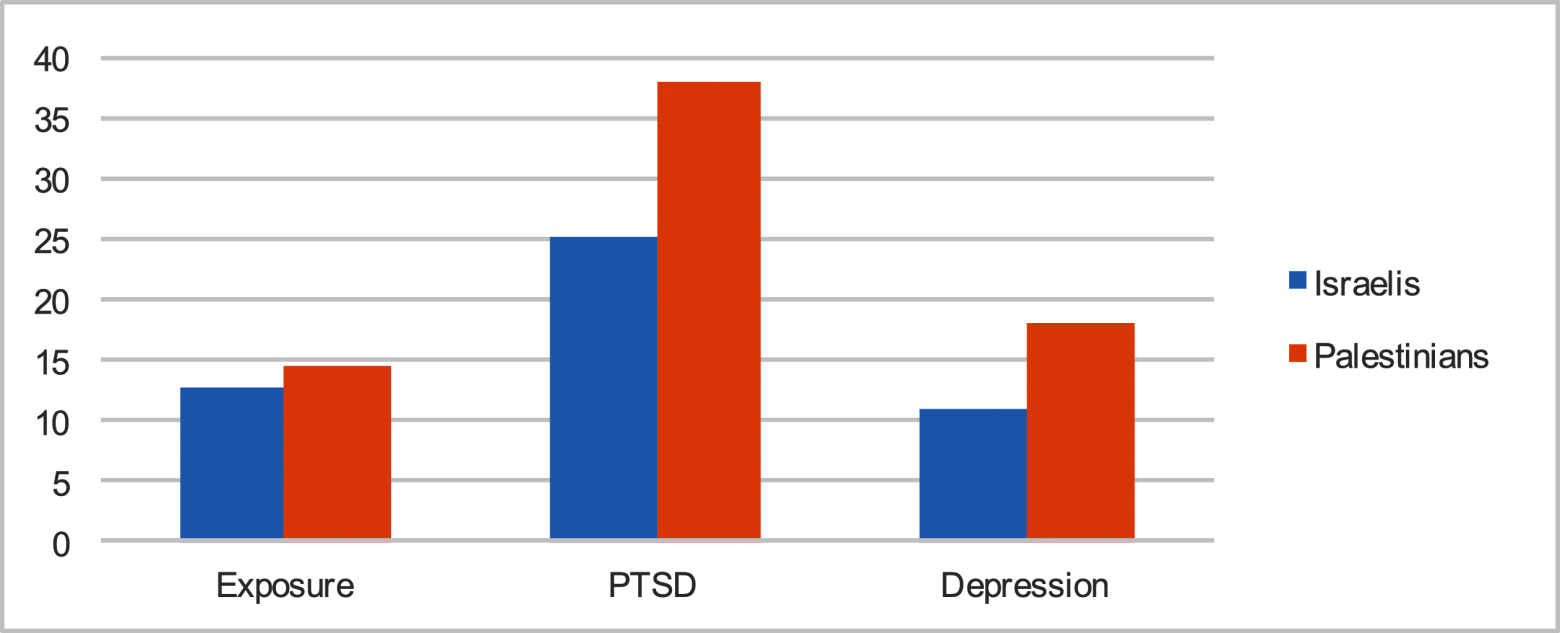
Psycho-emotional symptoms.

We examined the distribution of all research variables for the entire sample (see Fig 1). Most variables revealed normal curve distribution; however, three variables deserve further attention. First, exposure to trauma is found to be skewed to the right (range 0–131, M = 13.94, SD = 11.17). In other words, more respondents report lower than the average exposure to trauma and 14% report no exposure to traumatic events at all. Second, reported PTSD symptoms are also show a right skewed distribution (range 4–68, M = 33.58, SD = 11.85). Only 14% reported no PTSD symptoms or very low PTSD symptoms. Third, depression symptom distribution (range 6–36, M = 15.58, SD = 6.55) indicates that about 20% of the entire sample reports very low signs of depression, another 50% low levels, and about 30% medium to high levels, therefore, showing a right skewed distribution.

**Fig 2:**
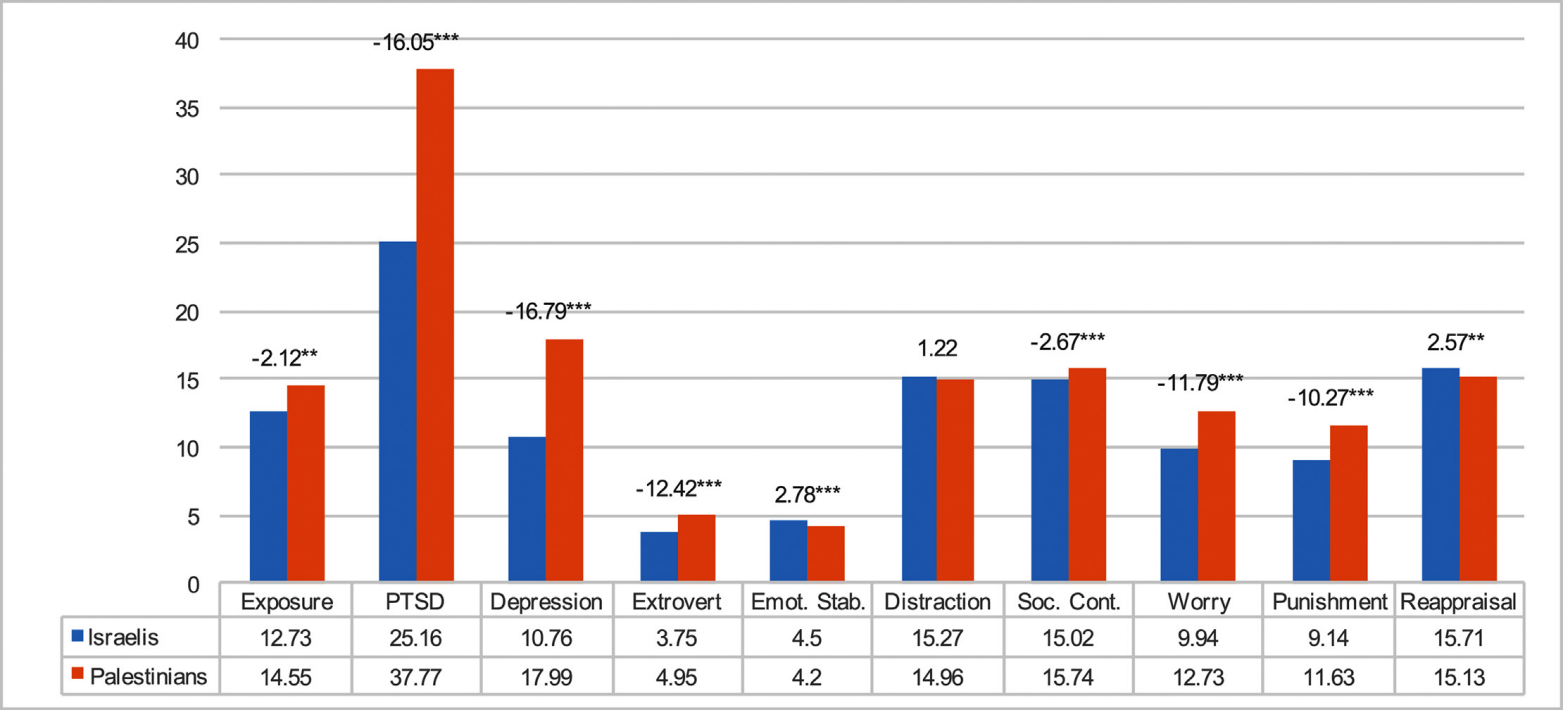
Differences between the two national groups on each of the research variables.

We examined the differences between Palestinians and Israelis on each of the research variables (Table 1 and Fig 2). Results indicated significant difference between the two groups with regard to all research variables, excluding distraction. Palestinians reported significantly higher levels of exposure to trauma, PTSD, extraversion, and greater use of thought control strategies including social control, worry and punishment. Palestinians report a significantly lower level of emotional stability and less frequent use of reappraisal. These results support our first hypothesis that Palestinian students will show higher levels of trauma experience, PTSD symptoms and depression compared with Israeli students. With a socioeconomic disparity between the two groups, the COR theory and our findings maintain the notion that correlations among our research variables are exacerbated due to Palestinians’ demographic condition and higher level of resources loss.

**Table 1:** Means, standard deviation and t-test for all research variables.

	Variable	Whole sample (n = 770) M (SD)	Israelis (n = 256) M (SD)	Palestinians (n = 514) M (SD)	Score range	t-test^a^
Biographic	Male (%)	38	32	40	—	
	Age (years)	22.54 (3.58)	25.9 (4.67)	20.9 (1.75)	—	21.68***
	Exposure to trauma	13.94 (11.17)	13.13 (12.11)	15.03 (10.88)	0–77	-2.12**
	Family income	2.95 (1.36)	3.08 (1.33)	2.89 (1.36)	1–5	1.83^*^
Stress Indicators	PTSD symptoms	33.58 (11.86)	25.29 (8.11)	38.05 (11.06)	4–68	-16.05***
	Depression	15.58 (6.55)	10.74 (4.23)	18.07 (6.17)	6–36	-16.79***
Personality Traits	Extraversion	4.55 (1.38)	3.75 (1.14)	4.96(1.31)	1–7	-12.42***
	Emotional Stability	4.30 (1.38)	4.46 (1.34)	4.20 (1.41)	1–7	2.78***
Thought Control Strategies	Distraction	15.06 (3.30)	15.28 (3.14)	14.96 (3.34)	6–24	1.22
	Social control	15.50 (3.49)	15.23 (3.23)	15.74 (3.33)	6–24	-2.67***
	Worry	11.78 (3.33)	9.92 (2.83)	12.71 (3.14)	6–24	-11.79***
	Punishment	10.80 (3.34)	9.16 (2.53)	11.59 (3.33)	6–24	-10.27***
	Reappraisal	15.32 (2.97)	15.72 (3.51)	15.12 (2.56)	7–24	2.57**

*p<0.1

**p<0.05

***p<0.01.

^a^Missing observations, if there were any, were dropped from analysis separately for each t-test.

We examined the association between the investigated variables by Pearson correlations separately, for each group (Table 2). Results of Pearson correlations for Israelis and Palestinians indicate: (a) the two dependent variables (PTSD and depression) significantly and positively correlate with each other (Israelis: r = .605, Palestinians r = .667, p < .001). Unsurprisingly, the more symptoms of PTSD an individual has, the more depressive symptoms they will also have. (b) There is a significant correlation between extraversion and emotional stability with PTSD symptoms and depression among only the Palestinians: The more introverted and less emotionally stable, the greater the levels of PTSD symptoms and depression (p < .001). (c) Worry and punishment significantly correlate with PTSD symptoms and depression among both groups: The more individuals resort to worry and punishment (thought control strategies), the higher the level of PTSD symptoms and depression (p < .001). (d) Three thought control strategies significantly correlate with depression among the Palestinians only: lower use of distraction, lower use of social control, and more use of reappraisal, result in higher levels of depression (p < .05). (e) Reappraisal correlates significantly with PTSD symptoms among the Palestinians only: The more use of reappraisal, the more PTSD symptoms (p < .001). It seems evident that personality factors and thought control strategies with PTSD and depression affect Palestinians and Israelis differently. In most of the analysis, results indicate, as hypothesized, that the correlations of both personality factors and thought control strategies with PTSD and depression are stronger among Palestinians.

**Table 2:** Bivariate correlations among research variables by group.

	1	2	3	4	5	6	7	8	9	10
1. PTSD	Isr.	.605***	.019	-.006	.077	-.035	.252***	.308***	.107	.379***
	Pales.	.667***	*-*.216***	*-*.343***	*-*.*043*	*-*.*050*	.310***	.360***	.163***	.294***
2. Depression	Isr.	—	.007	-.098	-.001	-.090	.183**	.347***	.021	.321***
	Pales.	—	*-*.196***	*-*.341***	-.110**	-.097**	.241***	.359***	.103**	.232***
3. Extrovert	Isr.		—	-.062	.115	.048	.074	.081	.072	.106
	Pales.		—	.195***	.*073*	.183***	*-*.*073*	-.113**	.*065*	*-*.*030*
4. Emotional	Isr.			—	.062	.080	-.025	-.064	.158**	-.023
	Pales.			—	.136***	.*056*	-.103**	*-*.251***	*-*.*001*	-.112**
5. Distraction	Isr.				—	.172***	.287***	.099	.199***	.063
	Pales.				*—*	.*008*	.304***	.190***	.160***	.*014*
6. Social control	Isr.					—	-.080	-.105	.327***	-.051
	Pales.					*—*	*-*.*081*	*-*.134***	.194***	.137***
7. Worry	Isr.						—	.355***	.200***	.127**
	Pales.						—	.499***	.177***	.*077*
8. Punishment	Isr.							—	.133**	.273***
	Pales.							—	.191***	.*027*
9. Reappraisal	Isr.								—	.117**
	Pales.								*—*	.099**
10. Exposure										—
										—

*p<0.1

**p<0.05

***p<0.01.

**Fig 3:**
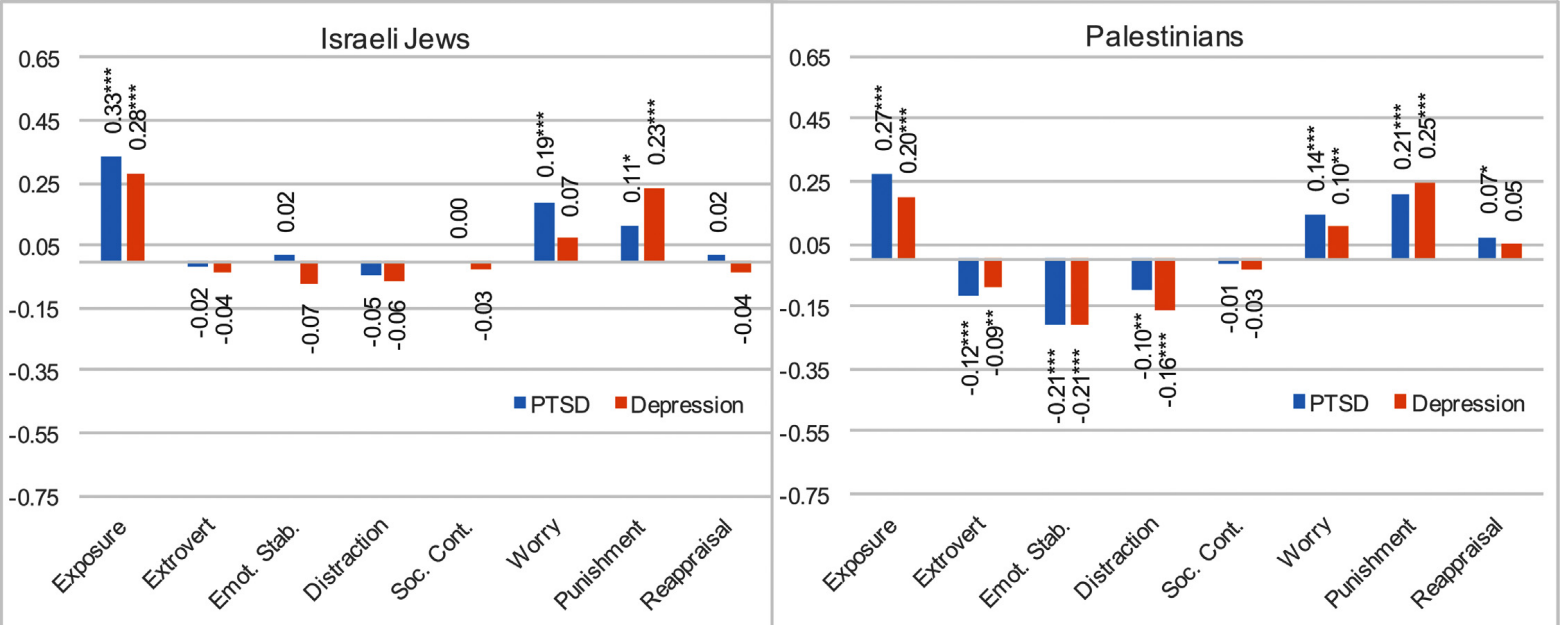
Standardized estimates for exposure, extraversion, emotional stability, distraction, social control, worry, punishment and reappraisal on PTSD and depression among Israeli Jews and Palestinians.

The unique prediction of exposure, namely that the two personality factors and the five thought control strategies link to PTSD symptoms and depression, were examined using separate path analysis [74] for each group (see Table 3 and Fig 3). Level of exposure to traumatic experience, extraversion, emotional stability, distraction, social control, worry, punishment and reappraisal constitute the predictors, and PTSD and depression symptoms are the dependent variables. All paths between the predictors and the dependents are examined (saturated model). The findings of this analysis revealed the relationship between several variables of interest.

**Table 3:** Path analyses with standardized estimates for exposure, extraversion, emotional stability, distraction, social control, worry, punishment and reappraisal on PTSD and depression.

Predictors^a^	Nationality^b^	Standardized beta	
		PTSD	Depression
Exposure	Israelis	.33***	.28***
	Palestinians	.27***	.20***
Extraversion	Israelis	-.02	-.04
	Palestinians	-.12***	-.09**
Emotional	Israelis	.02	-.07
	Palestinians	-.21***	-.21***
Distraction	Israelis	-.05	-.06
	Palestinians	-.10**	-.16***
Social Control	Israelis	-.00	-.03
	Palestinians	-.01	-.03
Worry	Israelis	.19***	.07
	Palestinians	.14***	.10**
Punishment	Israelis	.11*	.23***
	Palestinians	.21***	.25***
Reappraisal	Israelis	.02	-.04
	Palestinians	.07*	.05
R^2c^	Israelis	.21	.19
	Palestinians	.33	.28

*p<0.1

**p<0.05

***p<0.01.

(a) All correlations among the predictors were calculated. (b) Correlations between errors estimates of the dependent variables were calculated. (c) The model controlled for gender.

**Fig 4:**
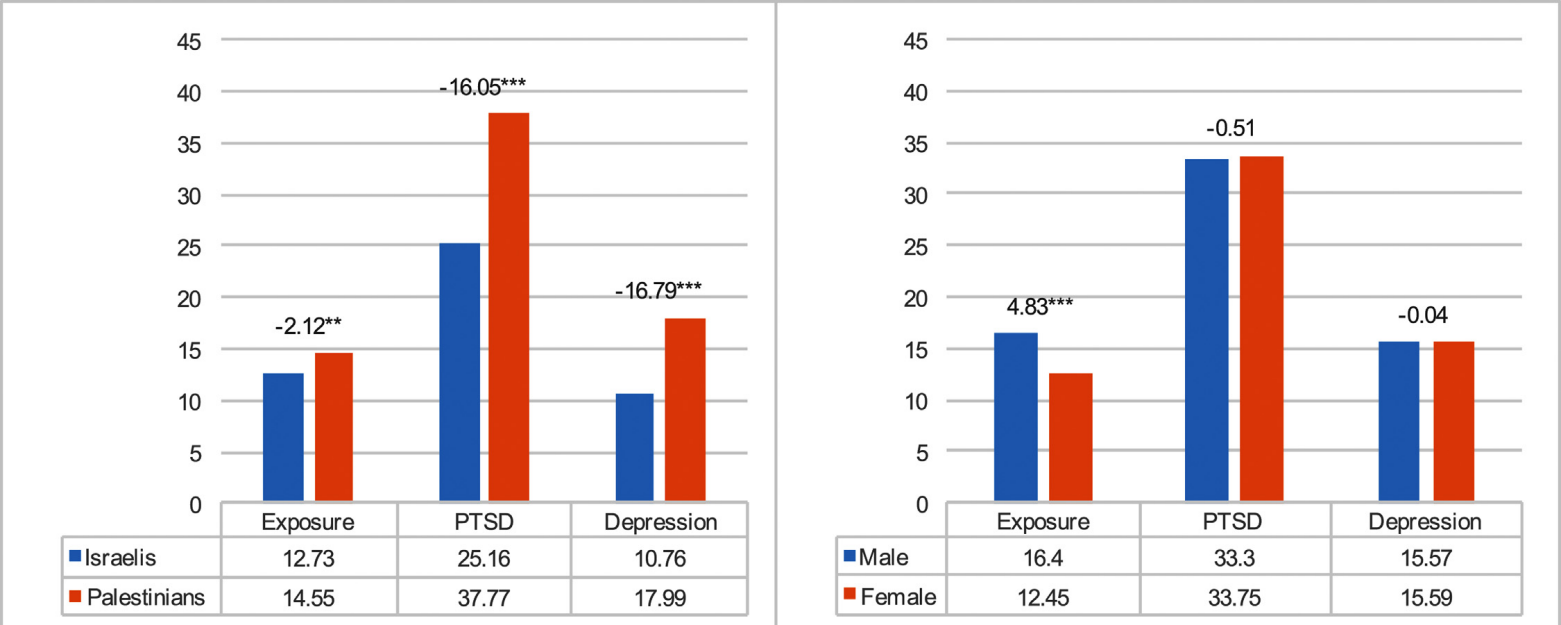
Means by nationality and gender.

From the context of our second and third hypotheses, we determined a number of conclusions. (a) Exposure to traumatic experiences significantly predicts PTSD and depression among Israelis and Palestinians: The more exposure, the higher PTSD symptoms and depression reported. (b) Extraversion and emotional stability significantly predict PTSD and depression only for the Palestinians: The less extraversion and less emotional stability, the more PTSD and depression. (c) The use of distraction as a thought control strategy significantly predicts PTSD and depression among only the Palestinians: the greater the use of distraction, the less PTSD and depression. (d) The use of worry as a thought control strategy significantly predicts PTSD and depression for Palestinians, and only PTSD for the Israelis: the greater the use of worry, the higher the level of PTSD and depression. (e) The use of punishment as a thought control strategy significantly predicts PTSD and depression among the Palestinians and Israelis: The more use of punishment, the higher level of PTSD symptoms and depression. The model for the Israelis explains 21% of the PTSD variance and 19% of the depression variance. The same model for the Palestinians explains 33% of the PTSD variance and 28% of the depression variance. These results largely support our hypothesis according which the five thought control strategies will significantly predict level of PTSD and depression, and these predictions will be stronger among Palestinians than Israelis (see Fig 4).

## Discussion

We find that personality factors can explain both PTSD and depression amongst civilians living in conflict zones. This particularly applies to groups with a lower socio-economic status and higher level of resources loss. Specifically, our work finds depression to be a common comorbidity of PTSD [20] and that prolonged exposure to conflict and political violence leads to high levels of PTSD and/or depression [21,32]. The findings that the Palestinians have higher rates of PTSD and depression corroborate previous findings that these conditions are exacerbated in poorer socio-economic societies [21, 75, 76].

We reveal that worry and punishment are positively correlated with PTSD across both groups [62, 63, 73]. We find that distraction, social control, and reappraisal correlate significantly negative with depression among Palestinians only (but not among Israelis): the more distraction, social control and reappraisal, the less depression. These results are somewhat unexpected since other studies reported that thought suppression were positively related to psychopathology [62]. One way to explain these results is to suggest that the Palestinian condition is much worse compared with the respondents in other studies. More research is needed to support our suggestion. Overall, the association between personality and thought control strategies with PTSD and depression is also more prevalent among Palestinians according to our hypothesis.

An additional way to explain our main results regarding personality and thought control and PTSD and depression, is to claim that prolonged exposure to conflict and political violence leads to high levels of PTSD and/or depression [21, 32]. Under these conditions the role of personality factors as well as thought control strategies, as predictors of PTSD and depression, become more significant. However, further studies are needed to support this explanation.

This study has several limitations to consider. Firstly, as any self-report study is subject to questions of objectivity, it could be that respondents give answers they believe the questioner wants to hear or which they think reflects well on themselves. Furthermore, though the Israeli university-going population is different in make-up to most Western countries, a similar study utilizing a wider sample of respondents from varying backgrounds would be beneficial.

Even so, our study shows that the variance between the Israeli and Palestinian results suggests that personality factors and exposure to trauma have a significant impact on the onset of PTSD and depression. The importance of personality factors and thought control strategies increase during traumatic events. More resources toward the healthcare sector and improved healthcare and social policies are required in order to provide support to those struggling with mental health issues and to provide the tools necessary for appropriate thought control strategies in order to minimize instances of PTSD and/or depression. These efforts could reduce the prevalence of threat perceptions and support for militant policies. Improving mental health is key to reducing violence; good mental health may provide the key to long-term conflict resolution.
